# Comparative Metabolic Defense Responses of Three Tree Species to the Supplemental Feeding Behavior of *Anoplophora glabripennis*

**DOI:** 10.3390/ijms252312716

**Published:** 2024-11-26

**Authors:** Ruohan Qi, Jiahe Pei, Quan Zhou, Keyu Hao, Yi Tian, Lili Ren, Youqing Luo

**Affiliations:** Beijing Key Laboratory for Forest Pest Control, Beijing Forestry University, Beijing 100083, China; qqqrh1999@bjfu.edu.cn (R.Q.); jiahepei@bjfu.edu.cn (J.P.); quan_z@bjfu.edu.cn (Q.Z.); keyuhao111@163.com (K.H.); ty200609@sina.com (Y.T.)

**Keywords:** Asian longhorned beetle, supplemental feeding, *Elaeagnus angustifolia*, plant defense, metabolites

## Abstract

*Elaeagnus angustifolia* L. can attract adult Asian longhorned beetle (ALB), *Anoplophora glabripennis* (Motschulsky), and kill their offspring by gum secretion in oviposition scars. This plant has the potential to be used as a dead-end trap tree for ALB management. However, there is a limited understanding of the attraction ability and biochemical defense response of *E. angustifolia* to ALB. In this study, we conducted host selection experiments with ALB and then performed physiological and biochemical assays on twigs from different tree species before and after ALB feeding. We analyzed the differential metabolites using the liquid chromatograph–mass spectrometer method. The results showed that ALB’s feeding preference was *E. angustifolia* > *P.× xiaohei* var. *gansuensis* > *P. alba* var. *pyramidalis*. After ALB feeding, the content of soluble sugars, soluble proteins, flavonoids, and tannins decreased significantly in all species. In three comparison groups, a total of 492 differential metabolites were identified (*E. angustifolia*:195, *P.× xiaohei* var. *gansuensis*:255, *P. alba* var. *pyramidalis*:244). Differential metabolites were divided into overlapping and specific metabolites for analysis. The overlapping differential metabolites 7-isojasmonic acid, zerumbone, and salicin in the twigs of three tree species showed upregulation after ALB feeding. The specific metabolites silibinin, catechin, and geniposide, in *E. angustifolia,* significantly increased after being damaged. Differential metabolites enriched in KEGG pathways indicated that ALB feeding activated tyrosine metabolism and the biosynthesis of phenylpropanoids in three tree species, with a particularly high enrichment of differential metabolites in the flavonoid biosynthesis pathway in *E. angustifolia*. This study provides the metabolic defense strategies of different tree species against ALB feeding and proposes candidate metabolites that can serve as metabolic biomarkers, potentially offering valuable insights into using *E. angustifolia* as a control measure against ALB.

## 1. Introduction

Plant-induced defense [1] refers to the response that plants generate after being damaged by insects [2,3]. This response includes producing defense proteins or toxic metabolites that can directly harm insects [4,5], achieving direct defense, or emitting volatiles to attract natural insect enemies for indirect defense [6,7]. The synthesis of secondary metabolites in plants is a crucial component of plant-induced defense [8,9]. In woody plants, coniferous trees frequently encounter insect damage, which prompts the production of traumatic resin ducts to enhance resin secretion [10,11] and the release of terpenoid volatiles [12], thereby protecting themselves. Additionally, some broad-leaved trees secrete gum [13,14] and accumulate flavonoids, alkaloids, and other substances for resistance [15,16,17], thereby reducing damage through these mechanisms.

*Anoplophora glabripennis* (Motschulsky, 1854) (Coleoptera: Cerambycidae), also known as the Asian longhorned beetle (ALB), is an invasive insect native to China and Korea. Since its first discovery in the United States in 1996 [18], it has invaded multiple countries in North America and Europe [19,20], causing significant damage to local broad-leaved trees [21,22]. The ALB is a wood-boring pest that feeds on a wide range of tree species, including *Populus*, *Salix*, *Acer*, *Ulmus*, and *Betula*, with a particular preference for the poplar tree [23,24,25]. The Asian longhorned beetle is characterized by a long life cycle and a concealed lifestyle, which contribute to the challenges in its control [26,27]. During their development, the larvae live within trees, feeding on the phloem and xylem [28]. As they emerge into adulthood, ALBs require nutrition from feeding on twigs [28]. Once they reach sexual maturity, the beetles engage in mating rituals. Following mating, the females carefully selected suitable host trees to create scars and deposit eggs [29,30,31]. The activities of adults, which included feeding, making scars, and laying eggs, all involved a selective and preferential process for host trees, further illustrating the beetle’s intricate relationship with its environment [32,33,34].

Although the ALB has a wide range of hosts, its preference for different tree species varies [35,36,37], and, similarly, different tree species exhibit varying resistance to ALBs [38,39]. Typically, some indicators are employed to assess the resistance of host plants to the Asian Longhorned Beetle, such as the number of oviposition scars, the number of frass holes, and the number of emergence holes [40,41]. In investigations into the categorization of host resistance levels to the ALB, it has been found that *Populus alba* var. *pyramidalis* Bge., *Fraxinus pennsylvanica* Marshall., and *Elaeagnus angustifolia* L. demonstrate a considerable degree of insect resistance and have been classified as resistant tree species [42,43,44]. Conversely, tree species susceptible to insect damage [45,46], such as *Acer negundo* L., *P.× xiaohei* var. *gansuensis* (C. Wang & H. L. Yang) and *Salix matsudana* Koidz., have been classified as susceptible tree species [47,48].

The concept of “dead-end trap plants” has been proposed in sustainable pest control research. Dead-end trap plants refer to a plant species that is highly attractive to pests but on which they cannot complete their development, reproduction, or survival [49]. The widely recognized example is that Vetiver (*Chrysopogon zizanioides* (L.) Roberty) can attract rice borers (*Chilo suppressalis* (Walker), *Semia inferens* (Walker), and *Busseola fusca* (Fuller)) to lay eggs [50,51,52]. In the rice-growing areas of southern China, Vetiver is used as a substitute for chemical pesticides for the comprehensive management of rice borers. In the northwest region of China, *E. angustifolia* exhibits a strong attraction to ALB, luring it to feed on twigs and lay eggs in the branches [53]. However, *E. angustifolia* secretes gum at the oviposition scars, which inhibits the eggs or the early instar larvae [54,55]. Consequently, based on the concept of dead-end trap plants, *E. angustifolia* can be considered a “dead-end trap tree” for the ALB [56], offering an ecological and sustainable approach to control the ALB population.

Much research [57,58,59,60] into the prevention and control of ALB has been conducted, but the use of *E. angustifolia* as a dead-end trap tree is currently in a blank stage. Therefore, our objective is to address three main issues: 1. What is the relative preference of ALB for selecting *E. angustifolia* compared to conventional host plants? 2. How do the soluble sugars, soluble protein, flavonoid, and tannin contents in different tree species change before and after feeding by the ALB? 3. How do different tree species defend against ALB feeding at the metabolic level? In this study, we selected three tree species, *P.× xiaohei* var. *gansuensis* (Y), *P. alba* var. *pyramidalis* (J), and *E. angustifolia* (S), as the subjects. We conducted feeding preference bioassays on the ALB in insect cages and collected plant tissues before and after feeding by the ALB. We quantified changes in their nutritional and secondary metabolite content and analyzed numerous metabolites using liquid chromatography-mass spectrometry (LC-MS). We aim to offer new insights and scientific evidence for the prevention and control of the Asian longhorned beetle.

## 2. Results

### 2.1. Feeding Preferences Among Different Tree Species of ALB Adults

After 48 h of feeding selection, it was found that the ALB had different preferences for various tree species, resulting in significant differences in feeding areas (Table 1). Specifically, it was observed that the ALB had a significant preference for *E. angustifolia* compared to the two species of poplar trees. In contrast, no significant difference in feeding preference was detected between *P.× xiaohei* var. *gansuensis* and *P. alba* var. *pyramidalis*. The results indicated that ALB adults prefer to feed on *E. angustifolia*, followed by *P.× xiaohei* var. *gansuensis*, and, finally, *P. alba* var. *pyramidalis*.

### 2.2. Nutrient and Secondary Metabolite Contents in Three Tree Species

**Figure 1:**
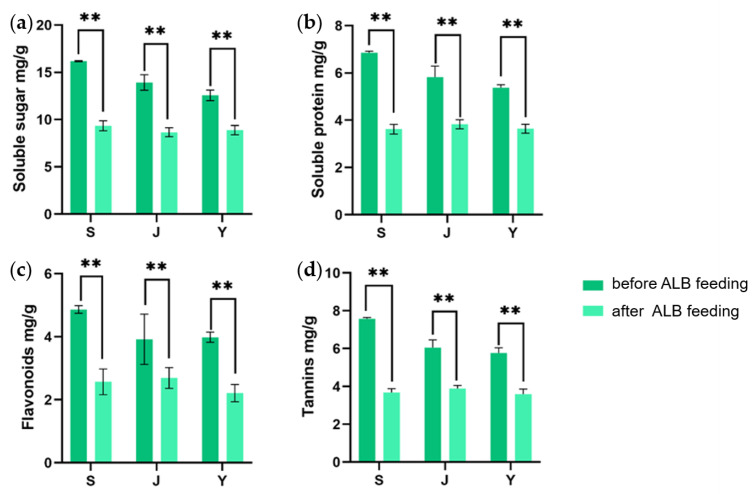
(**a**–**d**) Nutrient and secondary matter content of different trees after ALB feeding. Note: *E. angustifolia* (S), *P.× xiaohei* var. *gansuensis* (Y), *P. alba* var. *pyramidalis* (J) ** indicates statistically significant differences (*p* < 0.01).

Before ALB feeding, there were significant differences in the soluble sugar and soluble protein contents in the twigs of the three tree species, with the content in *E. angustifolia* being significantly higher than in the two poplar species (Figure 1a,b). Compared with healthy twigs, there were significant differences in the content of soluble protein and soluble sugars in the twigs after feeding (Figure 1a,b). After adult feeding, the soluble protein content in the twigs of *P. alba* var. *pyramidalis*, *P.× xiaohei* var. *gansuensis,* and *E. angustifolia* decreased by 43.76%, 32.34%, and 47.23%, respectively, while the soluble sugar content decreased by 37.79%, 29.28%, and 42.99%, respectively. Meanwhile, the extent of the decrease in soluble sugars and soluble proteins varied among the twigs of the three tree species, with the order being *E. angustifolia* > *P. alba* var. *pyramidalis* > *P.× xiaohei* var. *gansuensis*.

Similarly, there were significant differences in the content of flavonoids and tannins in the twigs of three tree species before feeding (Figure 1c,d). After adult feeding damage, the content of flavonoids and tannins in the twigs of the three tree species decreased. The flavonoid content decreased by 31.38%, 44.47%, and 47.12%, respectively, while the tannin content decreased by 35.87%, 37.78%, and 51.45%, respectively. The results (Figure 1) showed that the nutrient and secondary substance contents of twigs of all three tree species decreased after feeding by the ALB, but the rates of change were different. Among the three species, the substance content of *E. angustifolia* changed most significantly, both in nutrients represented by soluble protein and sugar and in secondary substances represented by flavonoids and tannins.

### 2.3. Identification and Analysis of Differential Metabolites Among Different Tree Species After ALB Feeding

**Figure 2:**
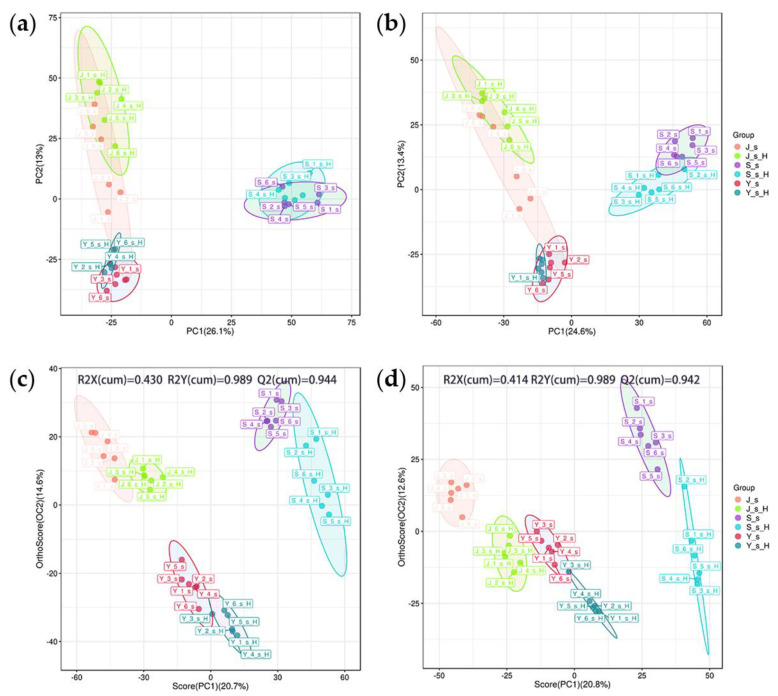
Dimensionality reduction analysis of differential metabolites. Note: (**a**,**b**): Principal Component Analysis (PCA); (**c**,**d**): Orthogonal Partial Least Squares Discriminant Analysis (OPLS-DA); positive ion mode on the left, negative ion mode on the right. *E. angustifolia* (S), *P.× xiaohei* var. *gansuensis* (Y), *P. alba* var. *pyramidalis* (J); J-s, S-s, and Y-s represent healthy twigs, and J-s-H, S-s-H, and Y-s-H represent twigs after ALB feeding.

We evaluated the data integrity of the mass spectrometry peaks extracted from all samples in both positive and negative ion modes. A total of 5264 peaks in positive ion mode and 4381 peaks in negative ion mode were retained, resulting in the identification of 757 metabolites. A total of 492 differential metabolites, 335 in the positive ion mode and 157 in the negative ion mode, were identified by screening with statistical tests using preset P-values and VIP and annotating the mass spectrometry data with five databases. PCA analysis showed that the samples were divided into three groups according to tree species (Figure 2a,b), indicating significant differences in metabolites between *E. angustifolia*, *P. alba* var. *pyramidalis,* and *P.× xiaohei* var. *gansuensis*. Further OPLS-DA results (Figure 2c,d) showed that there were significant changes in the metabolites within the twigs of the same tree species before and after feeding by the ALB.

**Figure 3:**
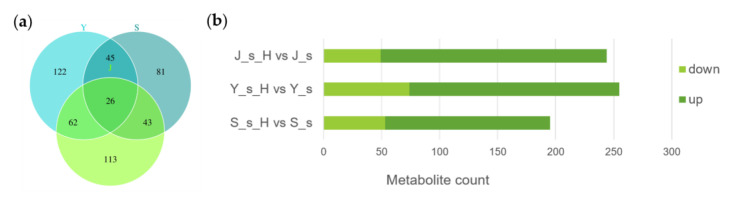
Statistical analysis of differential metabolites in three tree species twigs. Note: (**a**): Venn; (**b**): Bar chart. *E. angustifolia* (S), *P.× xiaohei* var. *gansuensis* (Y), *P. alba* var. *pyramidalis* (J); J_s, S_s, and Y_s represent healthy twigs, and J_s_H, S_s_H, and Y_s_H represent twigs after ALB feeding.

Healthy twigs were used as controls and compared with twigs fed by ALB to obtain differential metabolites (Figure 3a,b). Differential metabolite counts of *E. angustifolia*, *P. alba* var. *pyramidalis,* and *P.× xiaohei* var. *gansuensis* were 195, 244, and 255, respectively. Among them, there were 26 overlapping differential metabolites among the three. For two-by-two overlapping differential metabolites, there were a total of 45 for *E. angustifolia* and *P.× xiaohei* var. *gansuensis*, 43 for *E. angustifolia* and *P. alba* var. *pyramidalis*, and 62 for *P.× xiaohei* var. *gansuensis* and *P. alba* var. *pyramidalis*. Additionally, the distribution of specific metabolites among the three tree species was as follows: *E. angustifolia* had 81 metabolites, *P.× xiaohei* var. *gansuensis* 122, and *P. alba* var. *pyramidalis* 113. The number of up-regulated metabolites was generally much higher than the number of down-regulated metabolites after ALB feeding damage in all three species. We further analyzed the differential metabolites in the twigs of three tree species before and after infection, including overlapping and specific metabolites.

**Figure 4:**
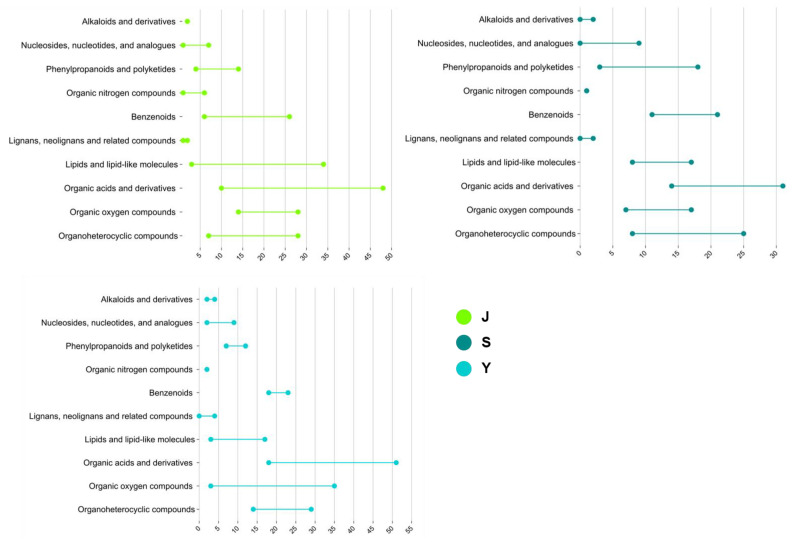
Classification of differential metabolites across different tree species. Note: *E. angustifolia* (S), *P.× xiaohei* var. *gansuensis* (Y), *P. alba* var. *pyramidalis* (J).

Based on the types and functions of the compounds, the differential metabolites were categorized into 10 categories, and, by combining the number of up- and down-regulated metabolites, the trends of different categories of differential metabolites could be clearly identified after ALB feeding. As shown in Figure 4, in *E. angustifolia*, the most changed metabolite type is Organic acids and derivatives, while the least changed metabolite type is Organic nitrogen compounds. In *P. alba* var. *pyramidalis*, the most changed metabolite type is also Organic acids and derivatives, but the least changed metabolite types are Lignans, neolignans, and related compounds. In the *P.× xiaohei* var. *gansuensis*, the most changed metabolites are Organic nitrogen compounds, and the least changed metabolites are Organic nitrogen compounds. After ALB feeding, the Organic acids and derivatives in the twigs of the three tree species exhibited the most significant changes, which may be part of the trees’ chemical defense against the ALB, indicating an induced resistance response to the insect feeding. Additionally, Alkaloids and derivatives, Phenylpropanoids and polyketides, Lignans, Neolignans, and related compounds, despite showing fewer changes, all belong to plant resistance substances, indicating an increase in the diversity of resistance substances in the twigs after ALB feeding.

**Figure 5:**
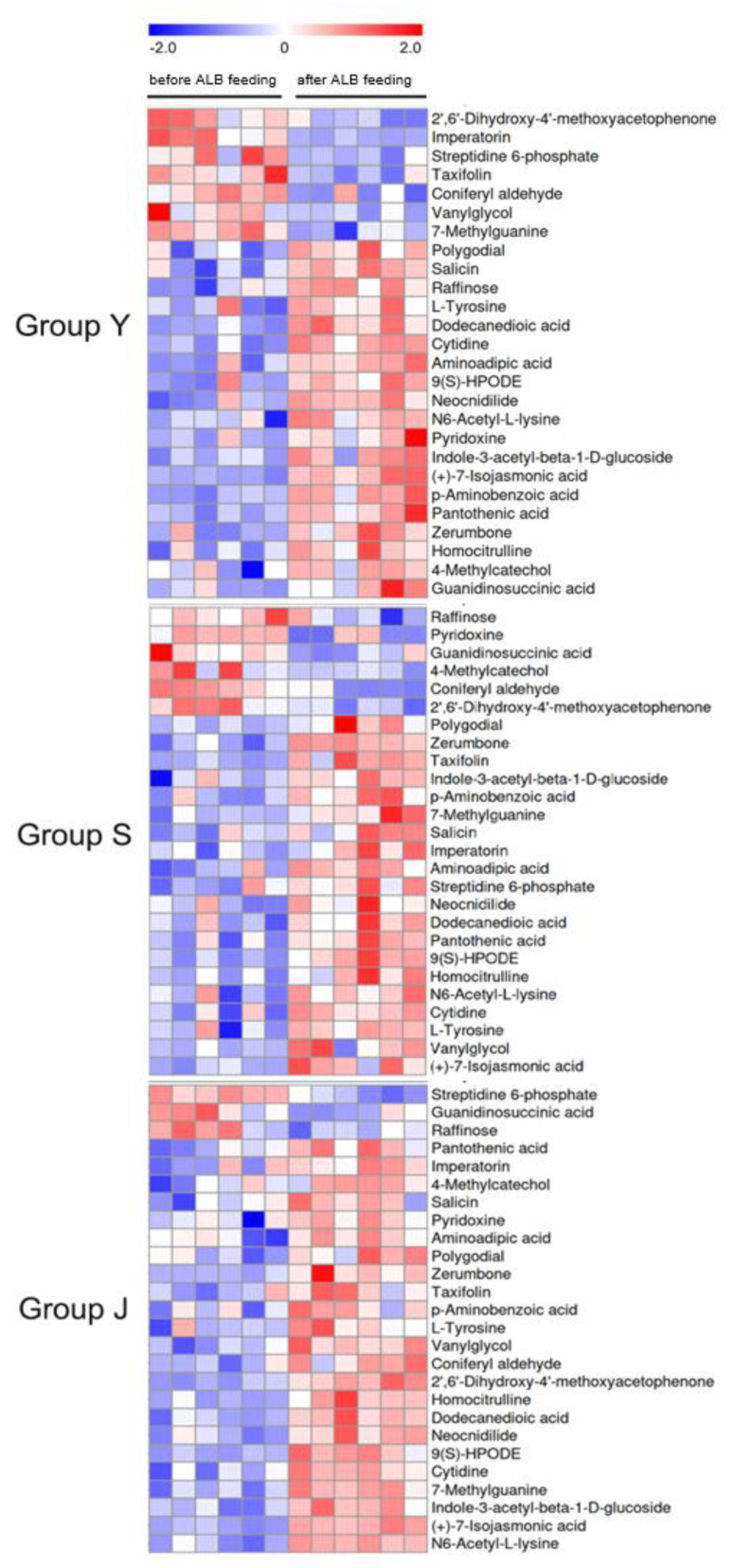
Cluster analysis of overlapping differential metabolites among twigs of three species. Note: *E. angustifolia* (S), *P.× xiaohei* var. *gansuensis* (Y), *P. alba* var. *pyramidalis* (J).

The contents of 26 overlapping differential metabolites varied among three tree species (Figure 5). After feeding by ALB, 19 metabolites were up-regulated in *P.× xiaohei* var. *gansuensis*, including Carbohydrates, Phenols, Nucleosides, Vitamins, Fatty Acids and derivatives, Amino Acids and derivatives, and Organic Acids and derivatives. Seven metabolites, 2’,6’-Dihydroxy-4-methoxyacetophenone, Imperatorin, Streptidine 6-phosphate, Taxifolin, Coniferyl Aldehyde, Vanylglycol, and 7-Methylguanine, were down-regulated. For *E. angustifolia*, 20 metabolites were upregulated by ALB feeding, including Phenols, Nucleosides, Fatty Acids and derivatives, Amino Acids and derivatives, and Organic Acids and derivatives. Six metabolites, Raffinose, Pyridoxine, Guanidinosuccinic Acid, 4-Methylcatechol, Coniferyl Aldehyde, and 2’,6’-Dihydroxy-4-methoxyacetophenone, were down-regulated. Metabolites of *P. alba* var. *pyramidalis* were upregulated after feeding by ALB, including Phenols, Nucleosides, Vitamins, Fatty Acids and derivatives, Amino Acids and derivatives, and Organic Acids and derivatives, and only three metabolites, Streptidine 6-phosphate, Guaniduccinic Acid, and Raffinose, were down-regulated. After ALB feeding, secondary resistance substances (Zerumbone, and Salicin) and JA derivatives of plant hormones ((+)7-isojasmonic acid) showed a consistent upregulation trend in twigs of the three species. However, Imperatorin and Taxifolin showed different changes in different tree species, with downregulation in *P.× xiaohei* var. *gansuensis*, but upregulation in both *P. alba* var. *pyramidalis* and *E. angustifolia.*

We screened differential metabolites specific to each tree species and selected the top five by |log2FC| (Table 2). As shown in the table, for the *P.× xiaohei* var. *gansuensis* group, after ALB feeding, the levels of Thymine, 6-Tuliposide B were up-regulated. In the *E. angustifolia* group, Silibinin, L-Leucine, Inosine, Catechin, and Geniposide were all up-regulated after ALB feeding. In the *P. alba* var. *pyramidalis* group, only Methyl 2-hydroxybenzoate and (+)-Fenchone were up-regulated. These findings suggested distinct metabolic responses in the twigs of the three tree species to ALB feeding, indicating species-specific metabolic adaptations and potential defense mechanisms against the pest.

### 2.4. KEGG Pathway Analysis of Differential Metabolites Induced by ALB Feeding

**Figure 6:**
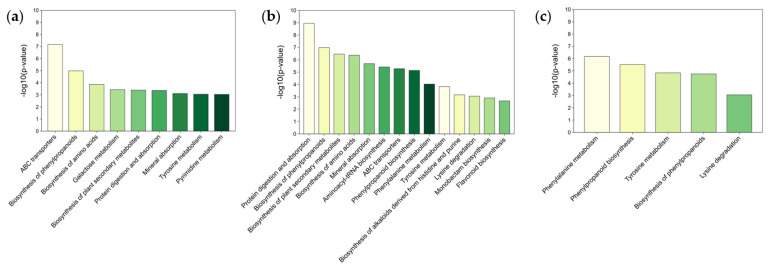
KEGG pathway analysis of different tree species. Note: (**a**) *P. alba* var. *pyramidalis*, (**b**) *E. angustifolia*, (**c**) *P.× xiaohei* var. *gansuensis*.

Differential metabolites were enriched in KEGG pathways, and pathways with a *p*-value less than 0.01 and a false discovery rate (FDR) less than 0.1 were selected to identify the differential metabolic pathways in the twigs of different tree species. A total of 14 pathways were identified in the *E. angustifolia*, 9 pathways in the *P. alba* var. *pyramidalis*, and 5 pathways in the *P.× xiaohei* var. *gansuensis* (Figure 6). Based on the previous results, differential metabolites were categorized into overlapping and specific, hence, the enriched pathways also included overlapping and specific pathways.

In the twigs of the three tree species, two overlapping metabolic pathways were significantly enriched, including Tyrosine metabolism and Biosynthesis of phenylpropanoids. *E. angustifolia* and *P.× xiaohei* var. *gansuensis* shared five enriched pathways, including Phenylpropanoid biosynthesis, Tyrosine metabolism, Biosynthesis of phenylpropanoids, and Lysine degradation (Figure 6b,c). *E. angustifolia* and *P. alba* var. *pyramidalis* shared seven enriched pathways, including ABC transporters, Biosynthesis of phenylpropanoids, Biosynthesis of amino acids, Biosynthesis of plant secondary metabolites, Protein digestion and absorption, Mineral absorption, and Tyrosine metabolism (Figure 6a,b). *P. alba* var. *pyramidalis* and *P.× xiaohei* var. *gansuensis* shared two enriched pathways, including Tyrosine metabolism and Biosynthesis of phenylpropanoids (Figure 6a,c). Additionally, specific metabolites were enriched in distinct metabolic pathways. P. alba var. pyramidalis was enriched in Galactose metabolism and Pyrimidine metabolism, while *E. angustifolia* was enriched in Aminoacyl-tRNA biosynthesis, Biosynthesis of alkaloids derived from histidine and purine, Monobactam biosynthesis, and Flavonoid biosynthesis. The results indicated that all three tree species, after ALB feeding, induced the Biosynthesis of phenylpropanoids and Tyrosine metabolism to accumulate more metabolites to cope with pest infestations. It also showed that *E. angustifolia* mobilized more pathways related to the synthesis of resistant substances after ALB feeding (Figure 6b).

## 3. Discussion

This study employed non-targeted metabolomics combined with physiological and biochemical indicators of the host twigs to investigate the metabolic responses of different host plants after ALB feeding.

In response to pest invasion, plants defend themselves by reducing their nutrient synthesis, thereby decreasing their attractiveness to insects [61,62]. As research has shown [63,64], plants rich in soluble sugars and proteins are better able to resist biological stress. We found that the soluble sugars and proteins in the twigs of *E. angustifolia* that were not affected by ALB were higher than those in the two species of poplar trees, indicating that *E. angustifolia* is better able to cope with pest damage than the two types of poplar trees. Our findings demonstrate a greater decline in resistant trees compared to susceptible ones following ALB feeding, corroborating previous studies [65,66]. Combined with the results of adult feeding preference, it could be inferred that the ALB prefers *E. angustifolia* due to its higher soluble sugars and soluble proteins. Plant secondary substances are commonly believed to inhibit the growth, development, and metabolism of insects [67]. Previous reports have found that the content of tannins in sweet oak (*Castanopsis eyrei* Tutch.) significantly increased when it experienced a mild level of harm after being consumed by *Aphrodisium faldermannii rufiventris* (Gressitt) [68]. Similarly, after feeding by *Batocera lineaolata* on different varieties of walnuts, the flavonoid content in the twigs increased [69]. However, in this study, the flavonoid and tannin contents of the three host twigs decreased after ALB feeding. We infer that the content of twigs after ALB feeding is dynamically changing and requires further temporal research. In this study, the significant upregulation of 7-isojasmonic acid in three tree species following ALB feeding suggests two key points. Firstly, it indicates the activation of defense pathways, as 7-isojasmonic acid is a precursor in the phenylpropanoid pathway, which is commonly associated with plant defense mechanisms [70]. This upregulation suggests that the plants are activating their defense pathways in response to ALB feeding. Secondly, it relates to the plant hormone JA, as 7-isojasmonic acid is the main metabolite in the 13 (S)-hydroperoxide linolenic acid (13-HPOT) metabolism pathway to generate 12 oxo-phytodienoic acid (OPDA), which is involved in defense against herbivores and necrotrophic pathogens [71]. Additionally, the varying responses of imperatorin, taxifolin, and coniferyl aldehyde across the three tree species can serve as metabolic biomarkers to assess the resistance of host twigs to insect feeding. Conversely, the discussion of species-specific differential metabolites may place greater emphasis on the significant changes unique to each tree species. The substance 6-Tuliposide B was upregulated in *P.× xiaohei* var. *gansuensis* twigs following ALB feeding, exhibiting antimicrobial activity to defend against pathogen invasion at the feeding wounds [72]. ALB feeding has induced changes in Methyl 2-hydroxybenzoate and (+)-Fenchone in *P. alba* var. *pyramidalis*, with these VOCs participating in the plant’s adaptation and defense mechanisms against biological stress [73,74]. The levels of Silibinin, Catechin, and Geniposide, all flavonoid compounds, were significantly altered in *E. angustifolia* in response to ALB feeding [75,76,77]. These findings may indicate that flavonoids in *E. angustifolia* may play a crucial role in defending against insect infestation.

Insect feeding interferes with the metabolism of the host plant, inducing changes in the plant’s metabolic patterns to cope with the disturbance and maintain normal activities [78,79,80]. KEGG pathway analysis across all tested tree species revealed the activation of conserved defense pathways, including Tyrosine metabolism and Phenylpropanoid biosynthesis, which were activated after ALB feeding, indicating the existence of conserved defense pathways [81,82]. The enrichment of Phenylpropanoid biosynthesis points to a shared strategy to produce resistance metabolites, like lignin and flavonoids, which are crucial for insect resistance [83,84]. Moreover, ALB feeding induced Tyrosine metabolism, boosting the synthesis of tocopherols, plastoquinones, and ubiquinones [85], providing precursors for other plant resistance pathways [86,87]. Specifically, *E. angustifolia* activates the Flavonoid biosynthesis and Biosynthesis of alkaloids derived from histidine and purine [88,89,90,91]. *P. alba* var. *pyramidalis* significantly induces Galactose metabolism and Pyrimidine metabolism following damage, generating substantial energy to combat pest invasion [92,93]. Based on the metabolic pathway results, we observed that *P.× xiaohei* var. *gansuensis* exhibits fewer enriched pathways for producing pest resistance substances, suggesting it may have lower resistance compared to *E. angustifolia* and *P. alba* var. *pyramidalis*.

## 4. Materials and Methods

### 4.1. Adults Feeding Preference Bioassays Among Three Tree Species

#### 4.1.1. Insect and Plant Materials

The adults of the Asian longhorned beetle were collected from an experimental site in Jiuquan City, Gansu Province, China. The original host was *P.× xiaohei* var. *gansuensis*. These adults were starved for 48 h before being used for feeding selection experiments. The three host trees twigs (*P.× xiaohei* var. *gansuensis*, *P. alba* var. *pyramidalis,* and *E. angustifolia*) used for feeding all come from Jiayuguan City, Gansu Province, China.

#### 4.1.2. Feeding Preference Behavior Bioassays

To explore the feeding preferences of ALB for different tree species, we selected fresh, one-year-old twigs, each approximately 60 cm in length and 1 cm in diameter [94]. Each tree species was set up with five biological replicates. The twigs were then randomly arranged in a plastic bucket with a diameter of 35 cm and a height of 50 cm. The bucket containing the twigs was placed in the center of a 100 cm × 100 cm × 100 cm insect cage. In each behavioral choice experiment, 6 adult beetles were placed in the center of the cage, with a total of 36 adult beetles (male:female = 1:1) tested. The twigs were collected 48 h after ALB feeding to calculate the feeding area. The feeding area was depicted on sulfuric acid paper and then outlined and calculated using ImageJ software ( version 1.53c) [95].

### 4.2. Nutrient and Secondary Metabolite Tests Content of Three Species Trees

#### 4.2.1. Sample Collection

Samples were collected from trees affected by ALB feeding in Jiuquan City, Gansu Province, China. These samples were obtained after 48 h of ALB feeding with the same site conditions and sampling times in order to eliminate the influence of other factors on the experimental materials. Healthy twigs were used as the control group. Each tree species was set up with six biological replicates. Samples were stored at −80 °C before use.

#### 4.2.2. Measurement of Nutrition and Secondary Metabolite Content

Soluble protein was measured using the BCA (Bicinchoninic Acid Assay) method [96], and the sample was prepared and placed under visible light at a wavelength of 562 nm for colorimetric analysis. The determination of soluble sugar content was carried out using the anthrone colorimetric method [97], and the sample was prepared and placed in the visible light region at a wavelength of 620 nm for colorimetric analysis. Tannins were determined using the phosphotungstic molybdic acid colorimetric method [98], and the samples were prepared and placed in the visible light region at a wavelength of 760 nm for colorimetric analysis. The flavonoid content was determined using the aluminum ion colorimetric method [99], and the absorbance value at 510 nm was used to calculate the flavonoid content in the sample. For all four substances, the measurements were conducted with six biological replicates.

### 4.3. Liquid Chromatography–Mass Spectrometry (LC-MS) Based Metabolite Profiling

Collected frozen twig samples were grounded to fine powder and weighed prior to extraction [100]. Metabolites were extracted from 50 mg of finely ground twig powder using 600 μL of methanol solution containing 4-chloro-L-phenylalanine (4 ppm). The LC analysis was performed on a Vanquish UHPLC System (Thermo Fisher Scientific, Waltham, MA, USA), and chromatography was carried out with an ACQUITY UPLC ^®^ HSS T3 (150 × 2.1 mm^2^, 1.8 µm) (Waters, Milford, MA, USA) [101]. Mass spectrometric detection of metabolites was performed on Q Exactive HF-X (Thermo Fisher Scientific, USA) with an ESI ion source. Simultaneous MS1 and MS/MS (Full MS-ddMS2 mode, data-dependent MS/MS) acquisition was used [102].

### 4.4. Data Processing and Statistical Analysis

Statistical analysis was carried out with the help of Tukey HSD and one-way ANOVA in the IBM SPSS Statistics for Windows, version 20.0 (IBMCorp., Armonk, NY, USA) [103] for the difference in the content of a substance between different samples. The raw data of metabolism were firstly converted to mzXML format by MSConvert in ProteoWizard software package (v3.0.8789) [104] and processed using XCMS package [105] for feature detection, retention time correction, and alignment. The metabolites were identified by accuracy mass (<30 ppm) and MS/MS data, which were matched with HMDB [106], massbank [107], LipidMaps [108], mzcloud [109], and KEGG [110]. The R software (version 4.4) package Ropls [111] was used to perform dimensionality reduction analysis on the sample data, calculate the *p*-value based on statistical tests, calculate the projection importance (VIP) of variables using the OPLS-DA dimensionality reduction method, and calculate the multiple of differences between groups using fold change. When *p* value < 0.05 and VIP > 1, metabolite molecules are considered statistically significant.

## 5. Conclusions

This study analyzed the feeding preferences of the Asian longhorned beetle (ALB) and the metabolic responses of three tree species, *E angustifolia*, *P.× xiaohei* var. *gansuensis*, and *P. alba* var. *pyramidalis*, to ALB’s supplemental feeding. Among the three tested tree species, *E. angustifolia* was the most favored host, followed by *P.× xiaohei* var. *gansuensis*, and *P. alba* var. *pyramidalis*. ALB feeding significantly reduced soluble sugars, proteins, flavonoids, and tannins in the hosts. Metabolomics identified 492 differential metabolites, among which the overlapping metabolites 7-isojasmonic acid, zerumbone, and salicin were induced in the twigs of all three tree species by the ALB. Significant changes in silibinin, catechin, and geniposide in *E. angustifolia* can serve as potential metabolic biomarkers for exploring defense mechanisms against ALB feeding. This research offers insights into the metabolic defense strategies of hosts against the ALB.

## Figures and Tables

**Table 1:** Feeding preferences of ALB on different tree species.

Species	Average Feeding Area/cm^2^
*P. alba* var. *pyramidalis*	0.50 ± 0.10 b
*P.× xiaohei* var. *gansuensis*	1.28 ± 0.32 b
*E. angustifolia*	3.00 ± 0.37 a

Note: Lowercase letters indicate significant differences (*p* < 0.05, one-way ANOVA followed by Tukey’s multiple comparisons test).

**Table 2:** Table of differential metabolites of different tree species after ALB feeding.

Species	Name	|log2FC|	−log10 (*p*.Value)	VIP	Regulation
*P.× xiaohei* var. *gansuensis*	Thymine	4.98	3.36	1.78	up
	Triphenyl phosphate	4.87	2.52	1.58	down
	Peonidin-3-glucoside	4.50	6.77	1.98	down
	(R)-4-Hydroxymandelate	4.48	2.46	1.69	down
	6-Tuliposide B	3.75	5.05	1.90	up
*E. angustifolia*	Silibinin	7.57	4.26	1.84	up
	L-Leucine	7.23	2.49	1.74	up
	Inosine	6.87	2.82	1.84	up
	Catechin	6.00	3.03	1.66	up
	Geniposide	5.67	2.67	1.60	up
*P. alba* var. *pyramidalis*	(+)-Fenchone	5.15	4.31	2.02	up
	Gentisic acid	4.26	2.49	1.64	down
	2-Heptanone	4.07	2.82	1.69	down
	Methyl 2-hydroxybenzoate	4.00	4.08	1.86	up
	Biliverdin	3.99	3.88	1.82	down

## Data Availability

The data presented in this study are available on request from the corresponding author.
